# Indirect selection on female extra-pair reproduction? Comparing the additive genetic value of maternal half-sib extra-pair and within-pair offspring

**DOI:** 10.1098/rspb.2011.2230

**Published:** 2011-11-23

**Authors:** Jane M. Reid, Rebecca J. Sardell

**Affiliations:** Institute of Biological and Environmental Sciences, School of Biological Sciences, Zoology Building, University of Aberdeen, Tillydrone Avenue, Aberdeen AB24 2TZ, UK

**Keywords:** breeding value, extra-pair reproduction, indirect selection, polyandry, quantitative genetics

## Abstract

One specific hypothesis explaining the evolution of extra-pair reproduction (EPR) by socially monogamous females is that EPR is under indirect selection because extra-pair offspring (EPO) sired by extra-pair males have higher additive genetic value for fitness than the within-pair offspring (WPO) a female would have produced had she solely mated with her socially paired male. This hypothesis has not been explicitly tested by comparing additive genetic value between EPO and the WPO they replaced. We show that the difference in additive genetic breeding value (BV) between EPO and the WPO they replaced is proportional to the genetic covariance between offspring fitness and male net paternity gain through EPR, and estimate this covariance with respect to offspring recruitment in free-living song sparrows (*Melospiza melodia*). Recruitment and net paternity gain showed non-zero additive genetic variance and heritability, and negative genetic covariance. Opposite to prediction, EPO therefore had lower BV for recruitment than the WPO they replaced. We thereby demonstrate an explicit quantitative genetic approach to testing the hypothesis that EPR allows polyandrous females to increase offspring additive genetic value, and suggest that there may be weak indirect selection against female EPR through reduced additive genetic value for recruitment of EPO versus WPO in song sparrows.

## 1. Introduction

The forces driving the evolution of extra-pair reproduction (EPR) by socially monogamous females, and of polyandry more generally, remain widely debated and unresolved [1–8]. One major hypothesis is that female EPR is under indirect selection because the extra-pair offspring (EPO) produced through EPR are fitter than the within-pair offspring (WPO) a female would have produced had she solely mated with her socially paired male [1–3]. This hypothesis is often implicitly genetic; in many socially monogamous but genetically polyandrous species, extra-pair (EP) sires do not obviously provide additional resources to a female's EPO. Any intrinsic difference in fitness between a female's EPO and WPO that could underlie indirect selection for female EPR is therefore postulated to reflect increased additive and/or non-additive genetic value of EPO, and hence increased value of the EP sire compared to the female's socially paired male with respect to one or both components of genetic variation (commonly termed ‘good genes’ and ‘compatible genes’ theories, respectively [1,2]). Most explicitly, if females produced EPO of higher additive genetic value for fitness than the WPO they replaced (reflecting higher additive genetic value of the EP versus within-pair (WP) sire) and there was additive genetic variation in female propensity for EPR, genes underlying female EPR could become correlated (in linkage disequilibrium) with genes underlying high paternal or offspring fitness. Female EPR could then evolve through indirect selection [3,9].

Such genetic covariances are difficult to measure, especially in free-living populations. One widely implemented short-cut to testing the broad hypothesis that female EPR is under indirect selection is therefore to compare the phenotypes of maternal half-sib EPO and WPO from the same clutch, brood or litter [2–4]. Such comparisons have the advantage that phenotypic effects of natal environment and maternal genes may be relatively similar across maternal half-sibs. Overall effects of paternity (and hence paternal genes) on offspring phenotype can therefore be estimated independent of environmental and maternal variation that can severely confound phenotypic comparisons among sires or broods [3,10]. Consequently, numerous field studies have compared phenotypic traits such as body size, immune response and early survival between maternal half-sib EPO and WPO from the same brood, and recent studies have compared major fitness components, including lifespan and lifetime breeding success [2,4,8,10–12]. Such comparisons are valuable in quantifying overall phenotypic differences between EPO and WPO, but face certain limitations in testing the specific hypothesis that females increase the genetic value of their offspring through EPR.

Explicit test of this hypothesis clearly requires the genetic values of WPO and EPO, rather than solely their phenotypic values, to be quantified and compared. Comparing phenotype, even between maternal half-sibs, could provide misleading conclusions regarding relative genetic value if environmental or maternal effects on individual phenotype covary with paternity within a brood or litter. For example, EPO may be conceived early within a brood, perhaps reflecting the timing of EP copulations [8]. Early offspring may also have higher fitness than later offspring independent of their paternity, owing to variation in competitive ability resulting from hatch or birth order within a brood. EPO may therefore be fitter than WPO because of their brood position rather than because they are of intrinsically higher genetic value [8,13,14]. The converse scenario, where EPO hatch later and have low fitness owing to their brood position, could also be postulated. Indeed, phenotypic comparisons between maternal half-sibs from the same brood do not control for any form of within-brood variation in environmental or non-genetic parental effects on offspring fitness in relation to paternity, whether owing to brood position or differential parental investment or any other mechanism [2,3,8,10,14]. Such effects could cause the difference in genetic value between EPO and WPO to be overestimated based on comparisons of phenotype, or underestimated if environmental effects systematically counteract genetic effects, providing misleading evidence regarding the benefit of EPR to polyandrous females.

This situation reflects a general limitation of testing the hypothesis that female EPR is under indirect selection by comparing phenotypes between observed maternal half-sib EPO and WPO. Strictly, the indirect selection hypothesis predicts that EPO will be fitter than the WPO a female would have produced had she solely mated with her socially paired mate (i.e. the WPO that the EPO replaced). It does not strictly predict that EPO will be fitter than the WPO a female produced anyway, despite having produced the EPO. However, the phenotypes of WPO that were not produced clearly cannot be measured and compared with the phenotypes of the EPO that were produced instead. The approach of comparing phenotypes between observed maternal half-sib WPO and EPO therefore requires an additional assumption: that the WPO that were produced are similar to the WPO that would have been produced if the female had not produced EPO instead. This may be valid (on average) with respect to genetic value, but it may not be valid with respect to environmental effects on phenotype, whether reflecting brood position or parental investment or any associated maternal effects.

A further constraint on comparing observed phenotypes between maternal half-sib EPO and WPO from the same brood or litter is that such analyses are necessarily restricted to mixed-paternity broods and cannot include broods that comprise entirely EPO (where EPR occurred to the degree that the female's socially paired male was completely cuckolded [10–12]). In these cases, there are no WPO whose phenotypes can be measured and compared with those of the EPO. Broods where all offspring were sired by an EP male, which may be important drivers of selection on EPR and highly informative in understanding the basis of such selection, are therefore excluded from key analyses.

Given the specific aim of testing the hypothesis that polyandrous females increase the genetic value of their offspring through EPR, these problems of distinguishing genetic and environmental effects underlying the relative fitness of WPO and EPO, comparing EPO with the WPO they replaced and therefore were not produced and incorporating broods that comprise entirely EPO can, in theory, be resolved by explicitly estimating quantitative genetic rather than phenotypic differences between maternal half-sib EPO and WPO . For example, an individual's breeding value (BV) for any trait, defined as the sum of the average additive effects of its alleles on that trait (hence measuring additive genetic value), can be estimated given sufficient phenotypic data from known relatives spread over environments and parents [15,16]. This approach allows BVs to be estimated for individuals whose own phenotype was not observed, as commonly done for sex-limited traits in individuals of the non-expressing sex. The same approach can, given sufficient observations of relatives' phenotypes, allow estimation of BVs for individuals that do not exist, such as the hypothetical WPO a female would have produced had she not produced EPO instead. The differences in BVs and phenotypic values between maternal half-sib WPO and EPO can then be compared, informing the degree to which differences in additive genetic value can be inferred from standard phenotypic comparisons.

Although quantitative genetic analysis potentially provides the most explicit test of the hypothesis that a female's EPO are of higher additive genetic value than the WPO they replaced, this approach imposes formidable data requirements, is vulnerable to bias and has not been implemented in a wild socially monogamous but genetically polyandrous population. We derived a general quantitative genetic expression describing the difference in BV between a female's EPO and the hypothetical WPO they replaced. We then used comprehensive genetic pedigree data from free-living song sparrows (*Melospiza melodia*) to parametrize this expression and explicitly test the hypothesis that a female's EPO have higher BV for survival to recruitment, one major fitness component, than the hypothetical WPO they replaced. We compared the estimated differences in sex-specific BVs and phenotypic values for recruitment between maternal half-sib EPO and WPO and consider the degree to which conclusions are consistent. We thereby formulate and implement an approach to explicitly comparing additive genetic value between EPO and the WPO they replaced, and discuss the contribution of additive genetic variation in offspring recruitment to indirect selection on female EPR.

## 2. Material and methods

### (a) Estimating the difference in breeding value

‘Animal models’ are generalized linear mixed models where pairwise coefficients of kinship (*k*) calculated from pedigree data define a matrix proportional to the variance–covariance matrix of additive genetic random effects [15,16]. They allow additive genetic variances (*V*_A_) and covariances (cov_A_) in and among phenotypic traits to be estimated given unbalanced data, inbreeding and selection [15,16]. They also allow BVs for focal traits to be estimated for all individuals included in the pedigree [15,16]. BVs for hypothetical individuals that did not exist (such as the WPO that EPO replaced) could therefore be directly estimated by coding these individuals in the pedigree, or calculated as half the sum of their parents' estimated BVs. The mean difference in BV between a female's EPO and the WPO they replaced (*E*[*Δ*BV]) could then be calculated.

However, estimated BVs may be biased if appropriate environmental effects on mean phenotype, environmental variances and genetic groups are not modelled, and error and uncertainty may be substantial if phenotypes are observed for only few relatives of each individual [17–19]. An individual's estimated BV can then largely reflect its own observed phenotype and consequently be biased by environmental or non-additive genetic sources of phenotypic variation. Furthermore, post hoc comparisons of estimated BVs among groups of individuals are likely to be biased if the groups are not specified in the underlying animal model, and associated statistical confidence is hard to estimate [18]. The most rigorous and unbiased approach is therefore to estimate *E*[*Δ*BV] directly within an animal model rather than by post hoc comparison of estimated BVs of EPO and WPO [18].

Direct estimation of *E*[*Δ*BV] requires this difference to be expressed as a genetic covariance between phenotypic traits that can be explicitly specified within an animal model. Algebraic transformation (electronic supplementary material) shows that *E*[*Δ*BV] = ½(cov_A_(*N*_E_ − *N*_C_,*W*)/*E*[*N*_E_]), where *W* is fitness, *N*_E_ is the number of EPO a male sired and *N*_C_ is the number of offspring that male lost through cuckoldry (the number of EPO produced by the male's socially paired female). The term (*N*_E_ − *N*_C_), therefore, describes a male's net paternity gain (or loss) through EPR, and *E*[*N*_E_] is mean *N*_E_ across all males. *E*[*Δ*BV] can therefore be estimated as half the genetic covariance between (*N*_E_ − *N*_C_) and *W* across all males, divided by *E*[*N*_E_]. *E*[*N*_E_] can be calculated directly from paternity data and the genetic covariance cov_A_(*N*_E_ − *N*_C_,*W*) can be estimated from a bivariate animal model for (*N*_E_ − *N*_C_) and *W*. The mean difference in BV between a female's EPO and the WPO they replaced can thus be estimated from a consistent model without post hoc analysis of estimated BVs. Positive *E*[*Δ*BV] would indicate that females' EPO had a higher BV for fitness than the hypothetical WPO they replaced, on average.

This basic expression for *E*[*Δ*BV] assumes that additive genetic effects on fitness are the same in males and females and hence that the inter-sex genetic correlation for *W* is *r*_mf_ ≈1. It also assumes that the genetic correlation between a male's net paternity gain through sons and daughters is *ca* 1. The latter constraint can be relaxed by considering net paternity gain through sons and daughters as separate traits. The mean difference in BV between a female's EP sons and the WP sons they are assumed to have replaced can be estimated as *E*[*Δ*BV_S_] = ½(cov_A_(*N*_ES_ − *N*_CS_,*W*)/*E*[*N*_ES_]), where *N*_ES_ and *N*_CS_ are the numbers of sons that a male gains and loses through EPR. An equivalent expression holds for daughters, such that *E*[*Δ*BV_D_] = ½(cov_A_(*N*_ED_ − *N*_CD_,*W*)/*E*[*N*_ED_]) (electronic supplementary material).

### (b) Study system

Direct estimation of *E*[*Δ*BV] requires *N*_E_, *N*_C_ and some measure of fitness *W* to be measured across numerous known relatives in a socially monogamous population with EPR. A long-term study of song sparrows on Mandarte Island, BC, Canada, fulfils these requirements.

Song sparrows of both sexes typically breed two to three times per season starting from age one year, and are primarily socially monogamous with well-defined social pairings [20]. Females incubate clutches (typically three to four eggs) and both socially paired parents provision hatched offspring [20]. However, they are genetically polygynandrous, with frequent EPR [21]. Mandarte (*ca* six hectares) holds a resident song sparrow population, recently numbering *ca* 15–45 breeding pairs, which has been studied intensively since 1975 [20]. Each year, all breeding attempts are closely monitored and all nests located. All chicks surviving to *ca* 6 days post-hatch are marked with unique combinations of coloured rings to allow individual identification. The occasional immigrants (1.1 yr^−1^ on average) are individually colour-ringed soon after arrival. This low immigration rate is sufficient to maintain allelic diversity [22] and prevent inbreeding from accumulating. All social parents of all offspring (those incubating clutches or provisioning chicks) are identified by observation. Owing to Mandarte's small size and the intensive fieldwork, the probability of resighting a surviving adult during any breeding season is effectively one. The local survival of all offspring from ringing at *ca* 6 days post-hatch to adulthood at age one year (hereafter ‘recruitment’) can therefore be documented with high confidence. The high local recruitment rate and absence of ringed sparrows breeding on surrounding islands suggest that emigration is relatively rare [12,20].

During 1993–2008, 99.4 per cent of ringed chicks and adults were blood sampled and genotyped at 13 polymorphic microsatellite loci to allow assignment of genetic parents [21]. Bayesian full probability models that incorporated genetic and spatial information allowed genetic sires to be assigned to 99.6 per cent of sampled offspring with at least 95 per cent individual-level confidence, and revealed that *ca* 28 per cent of hatched chicks were sired by a male other than a female's socially paired mate and hence were EPO (compared with 24 per cent in a nearby mainland song sparrow population [21]). The probability of excluding a female's social mate as sire was 0.9998. Approximately 44 per cent of broods contained at least 1 EPO at ringing. All genetic mothers matched those assigned by social behaviour [21]. Sampled chicks were sexed using standard molecular methods [23]. Molecular sexes matched those attributed from reproductive behaviour for all recruited individuals. Male net paternity gain (*N*_E_ − *N*_C_) through all offspring, and through sons and daughters separately, was calculated for each adult (≥ 1 year old) male alive in each year during 1993–2008.

### (c) Additive genetic (co)variances

The expression *E*[*Δ*BV] = ½(cov_A_(*N*_E_ − *N*_C_,*W*)/*E*[*N*_E_]) shows that non-zero *E*[*Δ*BV] requires non-zero cov_A_(*N*_E_ − *N*_C_,*W*). This, in turn, requires non-zero *V*_A_ in both (*N*_E_ − *N*_C_) and *W* (currently measured as offspring survival to recruitment), because *V*_A_ = 0 implies cov_A_ = 0. Verbally, the hypothesis that EPO and WPO differ in BV for recruitment requires that both recruitment and male net paternity gain through EPR show non-zero *V*_A_ and are heritable (*h*^2^ > 0 [1,9]). Therefore, to estimate *V*_A_ and *h*^2^ in these traits, we first fitted univariate animal models to phenotypic data describing whether or not each offspring ringed on Mandarte during 1993–2008 survived to recruit, and net paternity gain of all adult males alive in these years.

Recruitment models included fixed effects of an offspring's natal year and sex (because mean recruitment differs among cohorts and between males and females [20]), and a fixed regression on inbreeding coefficient (*f*, because recruitment shows inbreeding depression and failing to control for inbreeding depression can inflate estimates of *V*_A_ [24,25]). Models also included fixed effects of EP status (EPO versus WPO) to minimize the degree to which any environmental variation associated with status could confound estimated genetic effects [17,18] and a status by sex interaction (because relative recruitment differs between male and female EPO versus WPO in mixed-paternity broods [12]). However, estimates of *V*_A_ and *h*^2^ remained quantitatively similar when the latter two effects were excluded (electronic supplementary material). Random effects of an individual's natal brood, natal territory, mother identity and/or social father identity were initially fitted to estimate variances owing to consistent effects of brood, location and parents providing care [16,19]. However, these variances were close to zero and estimates of fixed effects, *V*_A_ and *h*^2^, remained quantitatively similar when they were excluded (electronic supplementary material). A bivariate animal model was then fitted to sex-specific recruitment data to estimate sex-specific *V*_A_, *h*^2^ and inbreeding depression in recruitment and the inter-sex genetic covariance (cov_mf_) and correlation (*r*_mf_), given sex-specific fixed effects of year, *f* and EP status.

Net paternity gain models included fixed effects of male breeding year, fixed regression on *f* and random effects of individual males to estimate ‘permanent individual’ variance (*V*_PI_) and account for repeated observations of individuals across multiple years. Random effects of a male's mother and social father identities were initially fitted, but estimated variances were close to zero and the estimates of inbreeding depression, *V*_A_ and *h*^2^ remained similar when these effects were excluded (electronic supplementary material).

Bivariate animal models for offspring recruitment and male net paternity gain were then fitted to estimate cov_A_(*N*_E_ − *N*_C_,*W*), with (*N*_E_ − *N*_C_) measured across all offspring, and across sons and daughters separately. Appropriate fixed and random effects were fitted as above.

### (d) Analysis implementation

Pedigree data for all offspring ringed during 1993–2008 were compiled from genetic parentage data, taking the most probable sire for each offspring [21,26,27]. Pedigree data for 1975–1992 were compiled from observed social parentage and combined with the 1993–2008 genetic data to provide a full pedigree for all individuals alive during 1975–2008. Assuming the unobserved EPR rate during 1975–1992 was similar to the 28 per cent observed during 1993–2008, and that all mothers were correctly assigned by social behaviour, *ca* 86 per cent of all 1975–1992 pedigree links will be correct. Using the 1975–1992 social pedigree data, even though uncorrected for EPR, is therefore likely to provide a more powerful analysis than simply using the corrected genetic pedigree data for 1993–2008 (thereby assuming zero relatedness among the 1993 breeders [26,27]). Inbreeding coefficients were calculated from the 1975–2008 pedigree using standard algorithms [24,26]. Kinship between immigrants and existing natives, and hence the *f* of offspring of immigrant–native pairings, was assumed to be zero relative to the baseline pedigree generation [24,28]. Inbreeding coefficients of immigrants are undefined relative to this baseline. Net paternity gain data from five immigrant males (with undefined *f*) were therefore excluded from analyses (electronic supplementary material).

Animal models were fitted using Bayesian methods implemented using MCMCglmm 2.14 in R v. 2.12.2 [29,30]. Recruitment was modelled as a binary trait with binomial errors, logit link and fixed residual variance *V*_R_ = 1 (by convention). Male net paternity gain was modelled assuming Gaussian errors (electronic supplementary material). Priors on fixed effects were normally distributed with mean zero and large variance (10^8^). Parameter expanded priors on variance components used normally distributed working parameter priors with mean zero and variance 1000 and inverse-Wishart distributed location effect priors with degree of belief and limit variance of one (forming a scaled non-central F-distribution [15,30]). Prior genetic covariances were specified as zero. Posterior distributions were robust to reasonable variation in prior specifications (electronic supplementary material). Residual covariances between male and female recruitment, and between recruitment and male net paternity gain, were fixed to zero. Analyses used ⩾2 005 000 iterations, burn-in ⩾5000 and thinning interval ⩾2000 to ensure low autocorrelation among thinned samples (less than 0.05). Posterior modes and 95% credible intervals (95% CI) for fixed effects, *V*_A_, *h*^2^, cov_mf_, *r*_mf_ and cov_A_(*N*_E_ − *N*_C_,*W*), were estimated across samples. The latent-scale heritability of recruitment was estimated as 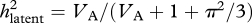 given *V*_R_ = 1 and logistic variance proportional to *π*^2^/3 [31]. 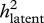 is interpretable as the genetic intra-class correlation, or as the heritability of a latent variable describing an individual's liability to recruit (where continuous variation is assumed to translate into recruitment at some threshold [15,31]). The probability-scale heritability of recruitment was estimated as 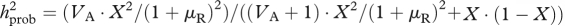, where *μ*_R_ is the mean recruitment rate and *X* = *μ*_R_/(1 + *μ*_R_) [31]. The heritability of male net paternity gain was estimated as 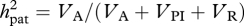. The inter-sex genetic correlation for recruitment was estimated as *r*_mf_ = cov_mf_/√(*V*_Am_ × *V*_Af_). The posterior distribution of the mean difference in BV between a female's EPO and the WPO they replaced (*E*[*Δ*BV]) was calculated by dividing the posterior distribution for cov_A_(*N*_E_ − *N*_C_,*W*) by 2*E*[*N*_E_].

### (e) Phenotypic variation

Our secondary aim was to quantify the degree to which the sex-specific difference in BV between EPO and the WPO they replaced would have been accurately estimated by comparing sex-specific phenotypic recruitment between EPO and WPO. However, the estimated difference in BV between EPO and the hypothetical WPO they replaced cannot be directly compared with the phenotypic difference between these individuals because hypothetical WPO have no observable phenotype. To estimate relevant phenotypic variation as well as feasible, we fitted a linear model with binomial errors and the same fixed effects as the univariate animal model to phenotypic recruitment data for all offspring hatched in broods that contained at least 1 EPO during 1993–2008.

## 3. Results

### (a) Recruitment

Survival from ringing to recruitment was observed for 2196 known-sex offspring hatched during 1993–2008, representing 807 broods (modal brood size 3, mean 2.72, range 1–4). These broods included 234 known mixed-paternity broods and 124 EPO-only broods at ringing. The 2196 offspring comprised 796 WP daughters, 312 EP daughters, 756 WP sons and 332 EP sons (totalling 1552 WPO and 644 EPO, and 1108 daughters and 1088 sons).

Overall, 413 (18.8%) of the 2196 offspring recruited on Mandarte. The pedigree, pruned to the 2196 offspring and all their known ancestors, comprised 2432 individuals. Mean pairwise *k* was 0.065 (median 0.061, inter-quartile range (IQR) 0.043–0.079) among all 2432 individuals and 0.069 (median 0.063, IQR 0.049–0.081) among the 2196 observed offspring (electronic supplementary material). Mean offspring *f* was 0.068 (median 0.064, IQR 0.038–0.090).

A univariate animal model fitted to recruitment data for all 2196 offspring showed significant *V*_A_ and *h*^2^ (table 1). There was substantial inbreeding depression such that more inbred offspring were less likely to recruit, and males were more likely to recruit than females (table 1). Phenotypic recruitment probability did not differ between EPO and WPO across all 2196 offspring, and the EP status by sex interaction was not significant (table 1 and electronic supplementary material).

**Table 1.** Posterior modes (and 95% credible intervals) for additive genetic variance, heritabilities and inbreeding depression in survival to recruitment, and fixed effects of sex and extra-pair (EP) status, estimated across 2196 known-sex song sparrow offspring in a univariate animal model. 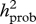 was estimated assuming *μ*_R_ = 0.19. Models without the EP status terms are provided as electronic supplementary material.

additive genetic variance (*V*_A_)	latent-scale heritability ( 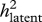 )	probability-scale heritability ( 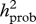 )	inbreeding depression (*β*_f_)	sex (male versus female)	extra-pair status (EPO versus WPO)	extra-pair status by sex interaction
0.61 (0.21 to 1.35)	0.13 (0.05 to 0.24)	0.07 (0.03 to 0.14)	−9.2 (−14.4 to −5.9)	0.37 (0.09 to 0.72)	−0.24 (−0.67 to 0.23)	0.11 (−0.36 to 0.85)

A bivariate animal model for male versus female recruitment showed that *V*_A_, *h*^2^ and inbreeding depression in recruitment were similar in both sexes and that *r*_mf_ ≈ 1 (table 2), indicating that genes underlying recruitment have similar additive effects in males and females. All offspring were consequently pooled in subsequent bivariate analyses to maximize statistical power.

**Table 2.** Posterior modes (and 95% credible intervals) for sex-specific additive genetic (co)variances, latent-scale heritabilities and inbreeding depression in survival to recruitment, the inter-sex genetic correlation and fixed effects of EP status, estimated across 1088 male and 1108 female song sparrow offspring in a bivariate animal model. The large 95% CIs compared with table 1 reflect reduced sample sizes and power when males and females are considered separately.

	additive genetic variance (*V*_A_)	latent-scale heritability ( 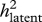 )	additive genetic covariance (cov_mf_)	genetic correlation (*r*_mf_)	inbreeding depression (*β*_f_)	extra-pair status (EPO versus WPO)
males	0.58 (0.07 to 1.90)	0.12 (0.03 to 0.32)	0.61 (0.14 to 1.37)	0.98 (0.85 to >0.999)	−8.9 (−15.5 to −3.9)	−0.15 (−0.44 to 0.41)
females	0.43 (0.09 to 1.75)	0.10 (0.04 to 0.31)			−9.7 (−16.0 to −3.7)	−0.22 (−0.67 to 0.26)

### (b) Male net paternity gain

Male net paternity gain (*N*_E_ − *N*_C_) was observed for 293 individual males totalling 738 male-years during 1993–2008. Of these males, 120 (41%) were observed in only 1 year (median 2 years, IQR 1–3, maximum 10). Net paternity gain varied from 11 to −11, 6 to −6 and 5 to −5 through all offspring, sons and daughters respectively (electronic supplementary material). The phenotypic correlation between net paternity gain through sons and daughters was 0.47 across all male-years. The pedigree, pruned to the 293 males and all their known ancestors, comprised 553 individuals. Mean pairwise *k* was 0.056 (median 0.052 and IQR 0.023–0.077) among all 553 individuals, and 0.071 (median 0.065 and IQR 0.048–0.085) among the 293 observed males. Mean male *f* was 0.057 (median 0.047 and IQR 0.029–0.075).

A univariate animal model fitted to male net paternity gain through all offspring indicated non-zero *V*_A_ and *h*^2^, although the 95% CIs converged towards zero (table 3). Net paternity gain tended to decrease with increasing male *f*, but the 95% CI for inbreeding depression overlapped zero (table 3).

**Table 3.** Posterior modes (and 95% credible intervals) for variance components, heritability and inbreeding depression in male net paternity gain (*N*_E_ − *N*_C_) estimated across 293 adult male song sparrows in a univariate animal model.

additive genetic variance (*V*_A_)	permanent individual variance (*V*_PI_)	residual variance (*V*_R_)	heritability ( 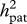 )	inbreeding depression (*β*_f_)
0.27 (<0.001 to 0.57)	0.001 (<0.001 to 0.24)	4.34 (3.78 to 4.70)	0.06 (<0.001 to 0.12)	−2.41 (−5.55 to 1.07)

### (c) Genetic covariance

The combined dataset comprising all adult males and offspring observed during 1993–2008 comprised 2266 individuals. The pedigree pruned to these 2266 individuals and all their known ancestors comprised 2445 individuals. Mean pairwise *k* was 0.065 (median 0.061 and IQR 0.043–0.079) among all 2445 individuals, and 0.068 (median 0.063 and IQR 0.048–0.081) among the 2266 individuals with phenotypic data.

Across all males and offspring, a bivariate animal model showed significant *V*_A_ and *h*^2^ in both recruitment and male net paternity gain (table 4). Furthermore, there was significant negative genetic covariance between the two (table 4). Because *E*[*N*_E_] = 0.85, the mean estimated difference in BV for recruitment between a female's EPO and the WPO they replaced was *E*[*Δ*BV] = −0.16 (95% CI −0.31 to −0.01). Estimates of cov_A_(*N*_E_ − *N*_C_,*W*) were also negative when analyses were repeated with net paternity gain through sons and daughters measured separately, although the 95% CI for sons overlapped zero (table 4). Because *E*[*N*_ES_] = 0.44 and *E*[*N*_ED_] = 0.42, the mean estimated difference in BV for recruitment between a female's EP sons and the WP sons they replaced was *E*[*Δ*BV_S_] = −0.15 (95% CI −0.32 to 0.04), and the corresponding difference for a female's EP versus WP daughters was *E*[*Δ*BV_D_] = −0.16 (95% CI −0.35 to −0.01). EPO therefore had a lower BV for recruitment than the WPO they replaced on average across all offspring, with similar estimated effects across sons and daughters. The estimate of *E*[*Δ*BV] ≈ −0.16 equates to an average reduction in phenotypic recruitment probability between maternal half-sib EPO and WPO of *ca* 0.02 in an average year.

**Table 4.** Posterior modes (and 95% credible intervals) for variance components, additive genetic covariance, heritabilities and inbreeding depression in male net paternity gain and offspring survival to recruitment estimated in a bivariate animal model. Net paternity gain was calculated across (*a*) all offspring (*N*_E_ − *N*_C_), (*b*) sons (*N*_ES_ − *N*_CS_) and (*c*) daughters (*N*_ED_ − *N*_CD_).

	additive genetic variance (*V*_A_)	permanent individual variance (*V*_PI_)	residual variance (*V*_R_)	heritability (*h*^2^)	additive genetic covariance (cov_A_)	inbreeding depression (*β*_f_)
(*a*) *N*_E_ − *N*_C_	0.23 (0.01 to 0.60)	0.001 (<0.001 to 0.24)	4.07 (3.76 to 4.63)	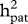 : 0.05 (0.01 to 0.12)	−0.27 (−0.53 to −0.02)	−0.90 (−5.12 to 1.15)
recruitment	0.61 (0.24 to 1.34)	—	1.0 (fixed)	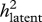 : 0.13 (0.06 to 0.24)		−9.7 (−14.5 to −5.8)
(*b*) *N*_ES_ − *N*_CS_	0.12 (0.02 to 0.27)	0.001 (<0.001 to 0.13)	1.56 (1.38 to 1.74)	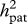 : 0.06 (0.01 to 0.15)	−0.14 (−0.28 to 0.03)	−0.33 (−2.54 to 1.71)
recruitment	0.70 (0.22 to 1.28)	—	1.0 (fixed)	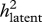 : 0.14 (0.05 to 0.23)		−9.9 (−13.7 to −5.4)
(*c*) *N*_ED_ − *N*_CD_	0.07 (<0.001 to 0.14)	0.001 (<0.001 to 0.06)	1.30 (1.16 to 1.44)	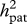 : 0.05 (0.01 to 0.10)	−0.14 (−0.29 to −0.01)	−1.25 (−3.32 to 0.34)
recruitment	0.80 (0.21 to 1.32)	—	1.0 (fixed)	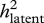 : 0.16 (0.06 to 0.24)		−9.3 (−14.0 to −5.5)

### (d) Phenotypic variation

**Figure 1.**
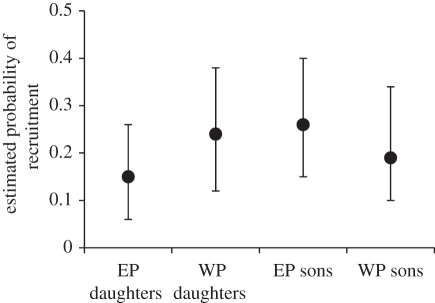
Posterior modal estimates (and 95% credible intervals (CIs)) for the phenotypic probability of recruitment of extra-pair (EP) and within-pair (WP) daughters and sons estimated across 1038 known-sex offspring hatched in 358 broods that contained at least 1 EPO. Posterior modes (and 95% CIs) for the main effects of inbreeding coefficient, EP status and EPO sex were −6.4 (−10.8 to −1.5), −0.58 (−1.10 to −0.01) and −0.32 (−0.84 to 0.36), respectively. The EP status by sex interaction was significant (posterior mode: 0.86, 95% CI: 0.05–1.58). The posterior modes for the sex-specific contrasts in recruitment probability between EPO and WPO were −0.08 (95% CI −0.18 to 0.01) and 0.05 (−0.04 to 0.15) for daughters and sons respectively.

A total of 1038 offspring hatched in 358 broods that contained at least 1 EPO at ringing, comprising 234 known mixed-paternity broods and 124 EPO-only broods. Across this restricted set of offspring, EP daughters tended to be less likely to recruit than WP daughters, while EP sons tended to be more likely to recruit than WP sons (figure 1). The EP status by sex interaction was significant and the estimated differences in recruitment probability were relatively large (figure 1). Although the 95% CIs for the sex-specific contrasts in recruitment probability between EPO and WPO overlapped zero, they did not overlap the posterior modal difference for the opposite sex (figure 1).

## 4. Discussion

One hypothesis explaining EPR by socially monogamous females is that a female's EPO are of higher additive genetic value than the WPO they replaced, and hence that EPR is under indirect selection through additive ‘good genes’ mechanisms [1–3]. This hypothesis differs from the hypotheses that EPR allows a female to produce additional offspring rather than solely replace WPO with fitter EPO (and is consequently under direct selection), and that EPR is under indirect selection through ‘compatible genes’ mechanisms [1,2,5]. However, the specific hypothesis that EPO are of higher additive genetic value than the WPO they replaced has not been explicitly tested in a wild population. The common short-cut approach of comparing phenotypes between observed maternal half-sib WPO and EPO from the same brood or litter is elegant in controlling for average phenotypic effects of environment and maternal genes but cannot distinguish paternal additive genetic effects from any individual environmental, parental or non-additive genetic effects associated with paternity, and inevitably excludes EPO-only broods where EPR occurred but no WPO are available for phenotypic comparison [2,8,10,13,14]. We suggest a direct approach to estimating the mean difference in additive genetic BV between EPO and the hypothetical maternal half-sib WPO they replaced (*E*[*Δ*BV]) by estimating the genetic covariance between fitness and male net paternity gain, and apply this approach to offspring recruitment in song sparrows.

### (a) Additive genetic (co)variances

Because *E*[*Δ*BV] is proportional to the genetic covariance between fitness and male net paternity gain, the existence of any indirect additive genetic benefit or cost of EPR through this route requires non-zero *V*_A_ in both fitness and net paternity gain. Indeed, there was significant *V*_A_ and *h*^2^ in offspring survival to recruitment, one major fitness component. This was evident in both males and females, with an inter-sex genetic correlation of *r*_mf_ ≈ 1. There was therefore some potential for female song sparrows to alter the additive genetic value for recruitment of their offspring through social and/or EP mate choice. Furthermore, there was evidence of non-zero *V*_A_ and *h*^2^ in male net paternity gain. Although 95% CIs converged towards zero in univariate analyses, estimates of *V*_A_ and *h*^2^ differed significantly from zero in bivariate analyses owing to increased power stemming from genetic covariance with recruitment. Indeed, the genetic covariance between net paternity gain and recruitment was significantly negative across all observed instances of EPR and across instances of EPR through daughters. The estimated effect was also negative across sons, although the 95% CI overlapped zero. Opposite to widespread prediction, a female's EPO therefore had lower BV for recruitment than the WPO they replaced on average, representing an average additive genetic cost of EPR to polyandrous females.

### (b) Phenotype versus breeding value

Across all 358 observed broods that contained at least 1 EPO at ringing, EP daughters tended to be less likely to recruit than WP daughters, while EP sons tended to be more likely to recruit than WP sons with a significant EP status by sex interaction and substantial estimated effects (figure 1). Similar variation was observed across offspring hatched in known mixed-paternity broods [12]. EP sires therefore tended to produce daughters but not sons with low phenotypic recruitment probability. Such phenotypic analyses cannot determine whether observed variation reflects additive genetic, non-additive genetic and/or environmental differences between EP and WP sons and daughters; yet these different mechanisms have very different implications for the evolution of female EPR [2,8,12]. Indeed, the estimated differences in mean BV for recruitment between EP and WP sons and daughters did not mirror observed phenotypic variation. Observed EP sons tended to be more likely to recruit than observed WP sons (by *ca* 7% on average), even though EP sons tended to have lower BVs than the hypothetical WP sons they replaced (equating to a *ca* 2% lower average genetic probability of recruitment). Observed EP daughters tended to be less likely to recruit than observed WP daughters (by *ca* 9%, on average), while EP daughters had a *ca* 2% lower average genetic recruitment probability than the hypothetical WP daughters they replaced. These discrepancies between estimated phenotypic and additive genetic effects should not be over-interpreted because BVs but not phenotypes can be measured for hypothetical WPO that did not exist, and the 95% CIs for the estimated sex-specific phenotypic effects overlapped the estimated genetic effects. However, the implication is that there may be sex-specific environmental, parental and/or non-additive genetic influences on the relative recruitment of EPO versus WPO [12]. One conceivable mechanism could be that EPO tend to hatch earlier within a brood than WPO [8,14] and early hatching benefits sons but not daughters. Female EPR, and hence phenotypic differences between EPO and WPO, could also arise because EP sires are less closely related to or otherwise more ‘compatible’ with a female than her socially paired male [1,2]. Substantial inbreeding depression was evident in offspring recruitment in both sexes, implying non-additive genetic variation (directional dominance) in recruitment (electronic supplementary material). A further conceivable mechanism could therefore be that females avoid inbreeding through EP sons more than daughters. These hypotheses remain to be tested. However, irrespective of the mechanisms, our analyses demonstrate that phenotypic comparisons among observed maternal half-sib EPO and WPO may not exactly mirror the sex-specific difference in BV between EPO and the WPO they replaced, either quantitatively or qualitatively. Such phenotypic comparisons should therefore be interpreted with some caution in the context of inferring indirect selection on female EPR through increased additive genetic value of EPO compared with their WPO maternal half-sibs [3,8,13,14].

### (c) Evolution of female extra-pair reproduction

A model predicting the rate of evolution of female EPR due to indirect selection through increased additive genetic value of EPO has been derived as 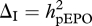 · *σ*_pEPO_ · *d*_EW,_ where *Δ*_I_ is the number of phenotypic standard deviations by which the mean EPR rate would evolve in one generation through such indirect selection alone, 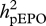 is the heritability of the proportion of a female's offspring that is sired by an EP male, *σ*_pEPO_ is the phenotypic standard deviation of this proportion and *d*_EW_ (strictly defined) is the difference in paternal additive genetic value for fitness across all EPO and WPO [3]. Values of 
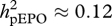
 and *σ*_pEPO_ ≈ 0.32 have been estimated for Mandarte's song sparrows [27]. The value of *d*_EW_ can be approximated from field studies as the mean difference in phenotypic fitness between maternal half-sib EPO and WPO [3]. However, *E*[*Δ*BV] may provide a better approximation because it eliminates any differential parental or environmental effects on phenotype. Taking *E*[*Δ*BV] for recruitment ≈ −0.16 gives an approximate point estimate of *Δ*_I_ ≈ −0.006. More comprehensive estimation of *Δ*_I_ using this approximation would require *E*[*Δ*BV] to be estimated for total fitness from conception rather than solely recruitment. This is challenging because the full distribution of fitness is difficult to measure and often highly skewed, and *r*_mf_ for total fitness may not be one. However, recruitment is a major determinant of fitness in song sparrows and other vertebrates [20,32]. Therefore, unless EPO have much higher additive genetic value for survival to hatch and/or reproductive success than the WPO they replaced, our current data indicate that *Δ*_I_ may be slightly negative in song sparrows, implying evolution towards less EPR. Other explanations for female EPR, including direct selection and other components of indirect selection, may therefore be required [2–6,26]. The observation that male net paternity gain (or loss) through EPR is significantly heritable is interesting in this context; genetic covariances between net paternity gain and other components of male and female fitness now need to be quantified.

### (d) The value of maternal half-sib comparisons

Comparing BVs between EPO and the hypothetical maternal half-sib WPO they replaced is insightful in explicitly indicating the additive genetic benefit that polyandrous females gain from EPR, including for EPO-only broods, and in considering the degree to which standard phenotypic comparisons between maternal half-sibs may accurately indicate this benefit. However, ultimately, the hypothesis that female EPR is under indirect selection through additive genetic mechanisms predicts and requires covariance between genes underlying female EPR and paternal and/or offspring fitness arising from within-pair and extra-pair reproduction across all polyandrous and monogamous females [3,9]. Estimation of this covariance has been simplified to a (phenotypic) comparison between half-sib EPO and WPO of polyandrous females purely for empirical tractability in wild populations [3,10]. However, EPO typically occur in a minority of broods in socially monogamous birds ([2]; *ca* 44% in Mandarte's song sparrows [21]). Even explicit comparisons of BV between maternal half-sib EPO and WPO may poorly indicate the overall genetic covariance between female EPR and offspring or paternal fitness if WPO-only broods occur non-randomly with respect to genetic values for female EPR, offspring and/or social male fitness (as might be hypothesized). WPO-only broods cannot contribute to phenotypic or quantitative genetic comparisons between observed or hypothetical maternal half-sib EPO and WPO because no EP sire exists or is known. Field studies aiming to dissect the selective forces driving female EPR, and definitively estimate *d*_EW_ and *Δ*_I_, must therefore go beyond the maternal half-sib comparisons that have become the default approach and estimate overall genetic covariances among EPR and paternal and offspring fitness.
